# Migrainin in Headaches

**Published:** 1903-01

**Authors:** 


### Migrainin in Headaches.

Migrainin is phenozon-caffein citrate and occurs as a white crystalline powder, readily soluble in water and alcohol. The solutions are slightly acid. It is used in migrain, headaches of influenza, in neuralgia, sciatica, following alcoholic excesses, nicotin poisoning, and the like.

In the treatment of migrain with phenacetin or antipyrin, the attack is delayed, while with migranin it is usually permanently stayed. In those attacks of typical hemicrania accompanied by gastric or gastrointestinal disturbances, anorexia, nausea, vomiting, meteorism, feeble and slow pulse, photophobia and general depression, amounting at times almost to unconsciousness, migrainin will invariably meet the indications without effect upon the pulse or heart. One author describes the action of migrainin as follows:

A few minutes after taking the remedy, a sensation of relief begins to make itself felt, as if the worst of the attack was passed. This assurance of relief becomes more distinct for the next quarter of an hour, frequently accompanied by a pleasing sensation of warmth, with a slight perspiration. In twenty or thirty minutes all pain has practically disappeared and complete recovery is effected within an hour, the general sensation of comfort lasting all day, and insuring a good night's rest.

For adults the dose of migrainin in all forms of headaches is 17 grains, preferably dissolved in a small glass of water, without additional quantity of water following. It should never be given in wine, beer, tea or other liquids. In general the patient should rest quietly for an hour and a half after the powder is taken. If necessary a second dose may be taken, but only in two hours. The remedy should be taken early, or directly after the first signs of headache appear and if possible on a empty stomach. Nothing should be eaten, nor drink of any kind for at least two hours after the remedy has been given. More than three powders in twenty-four hours are useless.—*Therapeutic Progress.*

